# Ppb-Level Hydrogen Sulfide Gas Sensor Based on the Nanocomposite of MoS_2_ Octahedron/ZnO-Zn_2_SnO_4_ Nanoparticles

**DOI:** 10.3390/molecules28073230

**Published:** 2023-04-04

**Authors:** Di Wu, Ali Akhtar

**Affiliations:** School of Information Science and Technology, Dalian Maritime University, Dalian 116026, China

**Keywords:** ZnO-Zn_2_SnO_4_ nanoparticles, MoS_2_ octahedron, hydrothermal method, n-n junction, gas sensor, H_2_S

## Abstract

Hydrogen sulfide (H_2_S) detection is extremely necessary due to its hazardous nature. Thus, the design of novel sensors to detect H_2_S gas at low temperatures is highly desirable. In this study, a series of nanocomposites based on MoS_2_ octahedrons and ZnO-Zn_2_SnO_4_ nanoparticles were synthesized through the hydrothermal method. Various characterizations such as X-ray diffraction (XRD), Brunauer–Emmett–Teller (BET), scanning electron microscopy (SEM), transmission electron microscopy (TEM), energy-dispersive X-ray spectroscopy (EDS) and X-ray photoelectron spectrum (XPS) have been used to verify the crystal phase, morphology and composition of synthesized nanocomposites. Three gas sensors based on the nanocomposites of pure ZnO-Zn_2_SnO_4_ (MS-ZNO-0), 5 wt% MoS_2_-ZnO-Zn_2_SnO_4_ (MS-ZNO-5) and 10 wt% MoS_2_-ZnO-Zn_2_SnO_4_ (MS-ZNO-10) were fabricated to check the gas sensing properties of various volatile organic compounds (VOCs). It showed that the gas sensor of (MS-ZNO-5) displayed the highest response of 4 to 2 ppm H_2_S and fewer responses to all other tested gases at 30 °C. The sensor of MS-ZNO-5 also displayed humble selectivity (1.6), good stability (35 days), promising reproducibility (5 cycles), rapid response/recovery times (10 s/6 s), a limit of detection (LOD) of 0.05 ppm H_2_S (R_a_/R_g_ = 1.8) and an almost linear relationship between H_2_S concentration and response. Several elements such as the structure of MoS_2_, higher BET-specific surface area, n-n junction and improvement in oxygen species corresponded to improving response.

## 1. Introduction

H_2_S gas, which has a rotten egg smell, could be considered one of the hazardous gases [1] which has an atrocious influence on human health. The long-term exposure to H_2_S gas at low concentrations (25–50 ppm) causes various diseases such as headaches, dizziness, nausea, vomiting and irritation in the eyes, etc. Moreover, high concentrations of H_2_S (more than 120 ppm) exposure may result in acute poisoning, paralysis and even sometimes death [2,3]. Thus, the veracious and real-time detection of H_2_S gas at low temperatures is very decisive, which is enabled by various semiconductor metal oxide (SMO)-based gas sensors.

Several gas sensors based on SMOs such as zinc oxide (ZnO), zinc gallate (ZnGa_2_O_4_), tin oxide (SnO_2_), nickel cobaltite (NiCo_2_O_4_), zinc stannate (Zn_2_SnO_4_), copper oxide (CuO), etc., possess some good properties such as precision, low cost, small dimensions, long-term stability and environmental friendliness; because of these properties, SMOs could be considered the primary candidates for the detection of toxic gases as well in photo-catalysis, etc. [4,5,6,7,8,9,10]. Plenty of research has shown that some complex metal oxides have been widely used as gas sensors in the last decades. Among these SMOs, an n-type SMOs Zn_2_SnO_4_ is an imperative ternary metal oxide with some properties such as high chemical stability and electron mobility, high conductivity, low visible adsorption, etc., have been studied widely in various fields such as photo-catalysis, solar cells and gas sensors [11,12,13]. Furthermore, ZnO, an n-type semiconductor material, has also been studied in various fields. The study described by An et al. showed that the sensor based on Zn_2_SnO_4_ detected the highest response to ethanol when compared with H_2_ [14]. Additionally, during the study on th sensing properties of ZnO nanosheets and nanorods, it was revealed that the sensor of ZnO nanosheets detected 100 ppm ethanol; by making comparison, it was noted that the response of nanosheets was 4.7-fold that of nanorods [15]. Some other sensors, such as Zn_2_SnO_4_-/ZnO-loaded Pd-based sensors, enhanced H_2_ sensing properties; the sensors based on the ZnO–SnO_2_–Zn_2_SnO_4_ hetero-junction, Pt–Zn_2_SnO_4_ hollow octahedron and Zn_2_SnO_4_/ZnO, revealed better gas sensing performances towards ethanol, acetone and formaldehyde, respectively [16,17,18]. However, pure metal oxides still face some demerits such as low response, high operating temperature, poor selectivity, etc. Therefore, their coupling with 2D materials is essential to increase the gas sensing properties of SMO-based gas sensors.

In the last few years, another research approach based on the emergence of 2D materials into SMOs has received great attention in various fields. Due to some rare properties such as narrow band gap, low density and thermal constancy, these have gained significant attention in the field of photo-catalysis, gas sensing, etc. [19,20,21,22,23,24]. Typical among transition metal dichalcogenides (TMDs), MoS_2_ has received gear attention as a fascinating candidate. This is not only as a gas sensor, but MoS2 can also be used for potential applications in the fields of photodetectors, solar cells, etc. [25,26,27]. Consequently, the synthesis of 2D material MoS_2_ is essential, which would accelerate the adsorption of oxygen molecules as well as increase gas sensing properties. For instance, various 2D materials and metal oxide-based gas sensors such as MoS_2_-reduced graphene oxide nanohybrid, MoS_2_@MoO_3_ magnetic hetero-structure, wool-based carbon fiber/MoS_2_ composite and ZnO-MoS_2_ nanocomposites were used to detect various types of VOCs, accompanied by high response, good selectivity, rapid response/recovery times, etc. [28,29,30,31].

The purpose of the current study was the detection of hazardous gases. In this regard, a series of gas sensors based on pure ZnO-Zn_2_SnO_4_ nanoparticles and octahedron MoS_2_ were synthesized via a simple hydrothermal method. To date, no literature has been reported on low-temperature gas sensors, such as 30 °C H_2_S gas sensors based on a MoS_2_-ZnO-Zn_2_SnO_4_ nanocomposite_._ Three gas sensors based on various nanocomposites (MS-ZNO-0, MS-ZNO-5, MS-ZNO-10) were tested to detect different hazardous gases, and our results studied that the highest response of 4 to 2 ppm H_2_S was received by the gas sensor of MS-ZNO-5. Furthermore, it revealed humble selectivity, good stability, rapid response/recovery times and LOD, promising reproducibility and an almost linear relationship between H_2_S concentration and response, suggesting its potential applicability in the field of gas sensors. Hence, the decoration of ZnO-Zn_2_SnO_4_ nanoparticles with octahedron MoS_2_ is expected to enable the generation of a novel sensor for H_2_S sensing.

## 2. Results and Discussion

### 2.1. Characterizations of Materials

**Figure 1:**
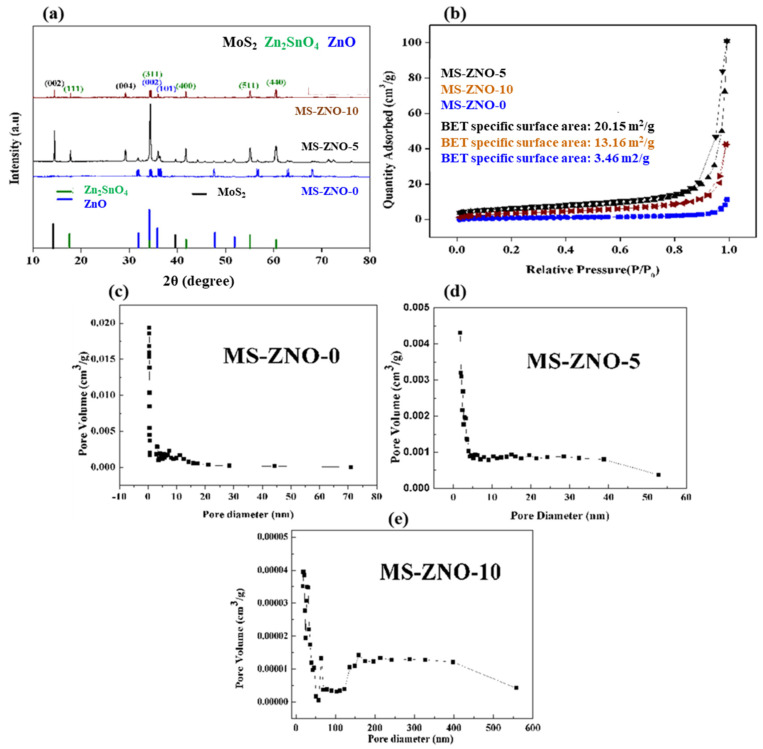
(**a**) XRD patterns; (**b**) N_2_ adsorption–desorption isotherms; (**c**) pore size distribution of MS-ZNO-0; (**d**) pore size distribution of MS-ZNO-5; (**e**) pore size distribution of MS-ZNO-10.

Figure 1a showed the XRD diffraction peaks of synthesized nanocomposites MS-ZNO-0, MS-ZNO-5 and MS-ZNO-10. The PDF numbers of Zn_2_SnO_4_, ZnO and MoS_2_ were PDF#24-1470, PDF#36-1451 and PDF#50-0739, respectively. Two diffraction peaks, cited at 2θ values of 34.42° and 36.25°, were matched well to the (002) and (101) crystal planes of ZnO. Furthermore, various peaks of Zn_2_SnO_4_ were found at 17.72°, 34.29°, 41.68°, 55.11° and 60.44°, and corresponded to the (111), (311), (400), (511) and (440) crystal planes of Zn_2_SnO_4_, respectively. The peak observed at half maximum (β) of the (311) and (002) was studied to check the average crystallite sizes. It was also notable that very fine peaks of MoS_2_ were also cited in both the nanocomposites (MS-ZNO-5 and MS-ZNO-10) at the 2θ values of 14.37 and 29.09, signified to the (002) and (004) crystal planes of MoS_2_. The estimated mean crystallite sizes such as 10.5, 22.5 and 12 of ZnO-Zn_2_SnO_4_ were examined by the Scherrer formula [32] in MS-ZNO-0, MS-ZNO-5 and MS-ZNO-10, respectively_._ The addition of MoS_2_ was reasoned to augment the crystallite sizes in both nanocomposites. Besides, the presence of MoS_2_ in composites was confirmed by other characteristics as well, such as SEM, TEM, EDS, XPS, etc. In order to check the BET-specific surface areas of three samples, N_2_ adsorption–desorption isotherms were studied in Figure 1b. The BET surface areas of MS-ZNO-0, MS-ZNO-5 and MS-ZNO-10 were 3.46, 20.15 and 13.16 m^2^/g, respectively. In addition, Figure 1c showed that the average pore sizes of MS-ZNO-0, MZ-ZNO-5 and MS-ZNO-10 were 11.1 nm, 8.1 nm and 11.3 nm, respectively.

**Figure 2:**
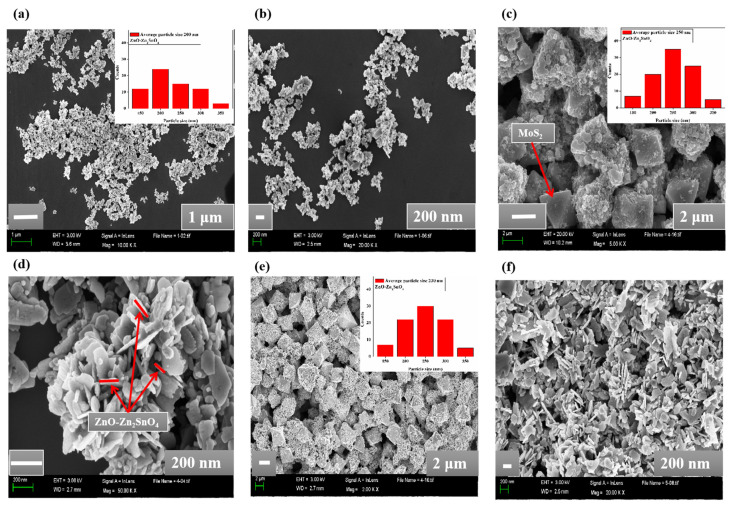
Morphologies of the nanocomposites: (**a**,**b**) SEM images of MS-ZNO-0; (**c**,**d**) SEM images of MS-ZNO-5; (**e**,**f**) SEM images of MS-ZNO-10.

As shown in Figure 2a–f, the morphology of the synthesized nanocomposites was observed from the SEM graphs. In Figure 2a,b, the SEM graphs of MS-ZNO-0 have been described. The ZnO-Zn_2_SnO_4_ nanoparticles, with an average particle size of 200–250 nm, were evaluated from the SEM images, while their modification with octahedron MoS_2_ was also confirmed from the SEM graphs of MS-ZNO-5 and MS-ZNO-10 in Figure 2c,d and Figure 2e,f, respectively. The SEM and TEM results showed that the relative particle sizes of ZnO-Zn_2_SnO_4_ nanoparticles and octahedron MoS_2_ were increased by the addition of MoS_2_ contents in the nanocomposite of MS-ZNO-5 but decreased a little in the nanocomposite of MS-ZNO-10, which also corresponds to the XRD results.

**Figure 3:**
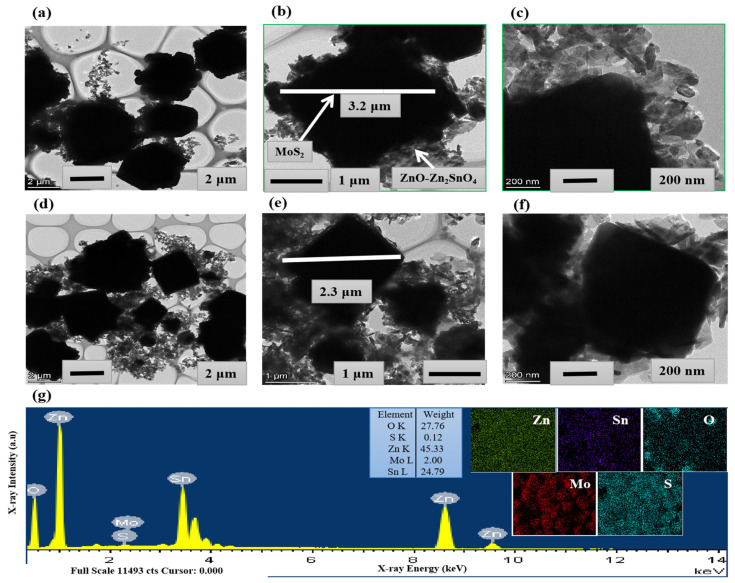
(**a**–**c**) TEM images of MS-ZNO-5; (**d**–**f**) TEM images of MS-ZNO-10; (**g**) EDS mapping and spectrum of all elements in MS-ZNO-5.

In Figure 3a–f, TEM results also proved the existence of MoS_2_ in nanocomposites of MS-ZNO-5 and MS-ZNO-10, with average particle sizes of 200−250 nm for ZnO-Zn_2_SnO_4_ nanoparticles in the nanocomposites, as shown below. The TEM graphs of MS-ZNO-5 in Figure 3a–f stated that particle size of ZnO-Zn_2_SnO_4_ nanoparticles was enhanced, while the size of octahedron MoS_2_ was almost 3.2 µm and 2.3 µmt in the TEM graphs of MS-ZNO-5 and MS-ZNO-10, respectively. The presence of octahedron MoS_2_ was further proved from the EDS spectrum of MS-ZNO-5. In Figure 3g, the EDS mapping images of Zn, Sn, O, Mo and S stated that each element had a uniform scattering effect in the nanocomposite of MS-ZNO-5.

**Figure 4:**
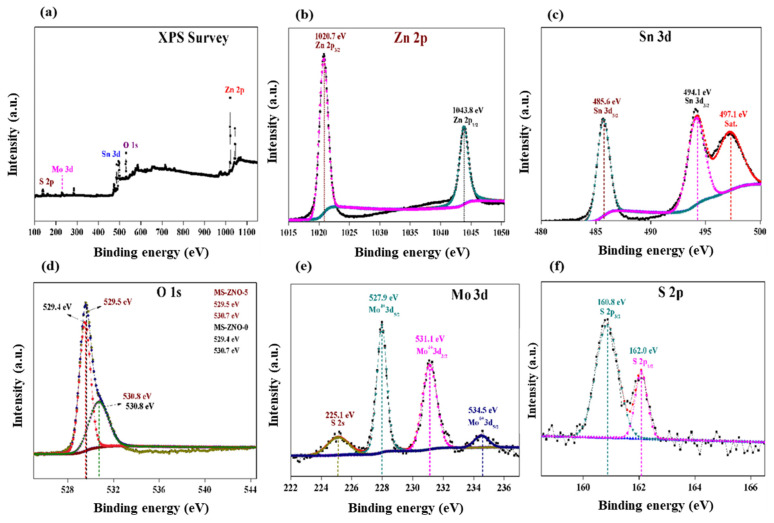
(**a**) XPS survey of MS-ZNO-5; (**b**,**c**) Zn 2p and Sn 3d spectrum’s of MS-ZNO-5; (**d**) O 1s spectrum of MS-ZNO-5 and MS-ZNO-0; (**e**,**f**) Mo 3d and S 2p spectrum’s of MS-ZNO-5.

The XPS data were fitted by XPSPEAK41 software. The X-ray photo-electron spectroscopy (XPS) results were revealed in Figure 4. The full XPS spectrum of the MS-ZNO-5 composite was disclosed in Figure 4a, which revealed the presence of all the elements such as Zn, Sn, O, Mo and S. In the XPS spectrum of Zn 2p (Figure 4b), two peaks, positioned at 1020.7 and 1043.8 eV, corresponded to Zn 2p_3/2_ and Zn 2p_1/2_ [33]. These results pointed out that Zn ions in the composite have a valence state of “2+”. In Figure 4c, the Sn 3d spectrum showed two peaks appearing at 485.6 and 496.1 eV corresponding to Sn 3d_5/2_ and Sn 3d_3/2_, respectively [34]. Additionally, one satellite peak at the value of 497.1 eV was cited. The O 1s spectrum of MS-ZNO-5 demonstrated more oxygen adsorption sites (O_V_), which was one of the factors required to enhance the gas sensing properties [8]. The peaks at the values of 529.5 and 530.8 eV in Figure 4d were matched to a typical metal–oxygen bond and defect sites in the XPS spectrum of MS-ZNO-5; on the contrary, in the XPS spectrum of MS-ZNO-0, the two peaks were cited at the values of 529.4 and 530.8 [35,36]. In the high-resolution spectra of Mo 3d, four peaks were cited. The peaks at the values of 225.1, 227.9, 231.1, 234.4 and 283.8 eV, shown in Figure 4e, were related to S 2s, Mo^4+^ 3d_5/2_, Mo^4+^ 3d_3/2_, and Mo^6+^ 3d_5/2_, respectively [37]. In Figure 4f, the peaks at the values of 160.8 eV and 162.0 eV were related to S 2p_3/2_ and S 2p_1/2_, respectively, in MoS_2_ [37].

### 2.2. Gas Sensing Properties

**Figure 5:**
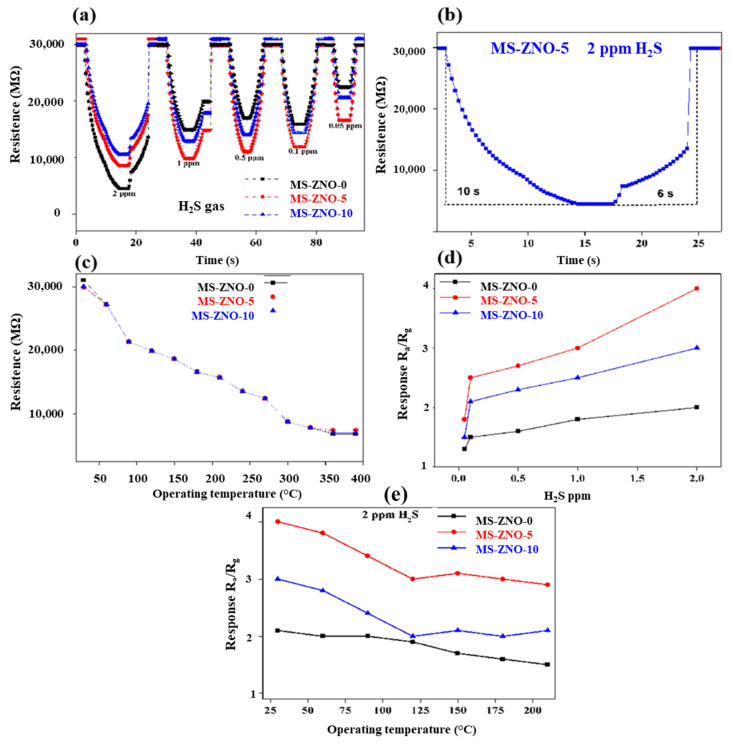
(**a**) Dynamic responses of all sensors to H_2_S 0.05-2 ppm at the operating temperature of 30 °C; (**b**) response/recovery diagram of MS-ZNO-5 towards 2 ppm H_2_S; (**c**) temperature resistance of pure and composite materials towards 2 ppm in the air at 30 °C; (**d**) graph of the relationship between the different concentration of H_2_S ppm and response at 30 °C; (**e**) the responses of all sensors towards 2 ppm H_2_S at various operating temperatures.

In this portion, the gas sensing properties of various sensors were studied. Their deep explanation was as follows: the response/recovery time curves for MS-ZNO-0, MS-ZNO-5 and MS-ZNO-10 were depicted in Figure 5a, which showed that response/recovery times for 2 ppm H_2_S were 10 s/6 s, (Figure 5b). Importantly, the minimum response of 1.8 to 0.05 ppm H_2_S was detected. The modification of octahedron MoS_2_ with ZnO-Zn_2_SnO_4_ nanoparticles not only enhances the BET surface area but also intensifies the adsorption of H_2_S molecules on the surface of the material, and it may also facilitate the adsorption of the oxygen species. These can be some factors which increase the gas sensing properties of nanocomposite (MS-ZNO-5). In Table 1, the gas sensing properties of the current sensor and some previous sensors were studied, which stated that the sensor based on MS-ZNO-5 received lower temperature gas sensing, better response, humble selectivity, LOD and rapid response/recovery times. Figure 5c was the temperature–resistance diagram of MS-ZNO-0, MS-ZNO-5 and MS-ZNO-10 at the operating temperature of 30 °C in air. When the operating temperature increased, the resistance showed a downward trend; this is because, when heating, the O^2−^ on the surface of both materials adsorbed oxygen and O^2−^ was converted into O^−^, which produces a large number of free electrons. At the same time, a large number of electrons reduced the concentration of negative ions on the surface of all the materials; in this way the potential barrier was twisted and the resistance was finally reduced [38,39]. As the temperature continued to enhance, the resistance of materials continued to decrease as the electrons moved faster. Figure 5d described the response vs. temperature curves, which specified that the response was improving with the increase in H_2_S concentrations. Thus, due to the linearity of the current sensor, it could be considered a promising material because of the linear relationship between H_2_S ppm and response. In Figure 5e, three sensors based on synthesized nanocomposites were tested to 2 ppm H_2_S at different operating temperatures. The maximum response detected by the sensor of MS-ZNO-5 was 4 to 2 ppm H_2_S at 30 °C. The decrease in response at higher temperatures could be explained as follows: the chemical activity is low at lower operating temperature, and vice versa. As a result, more gas molecules adsorbed onto the material surface quickly at higher temperatures, resulting in a decrease in response [40].

**Figure 6:**
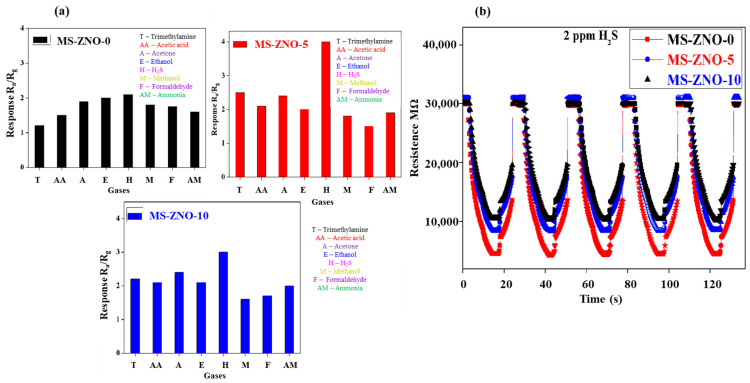
(**a**) Responses of MS-ZNO-0, MS-ZNO-5 and MS-ZNO-10 towards different gases (2 ppm); (**b**) reproducibility graphs of MS-ZNO-0, MS-ZNO-5 and MS-ZNO-10.

Figure 6a stated that the highest response to 2 ppm H_2_S was 4 and the second highest response to 2 ppm TMA was 2.5 at the operating temperature of 30 °C; in this way, the humble selectivity (S_2 ppm H2S_/S_2 ppm TMA_ = 1.6) was detected. The results about the reproducibility in Figure 6b demonstrated that the sensor based on MS-ZNO-5 showed promising reproducibility; it was checked around five times at the operating temperature of 30 °C and a similar response was noted. While other sensors detected good reproducibility, our main concern was the gas sensing properties of MS-ZNO-5. For this reason, the promising reproducibility was studied in correspondence with the sensor of MS-ZNO-5. The improvement in gas sensing response corresponded to some aspects such as the octahedron structure of MoS_2_; this enhanced BET surface area, allowing more oxygen species to adsorb onto the surface of the material and make the reaction faster to increase the gas sensing response [17].

**Figure 7:**
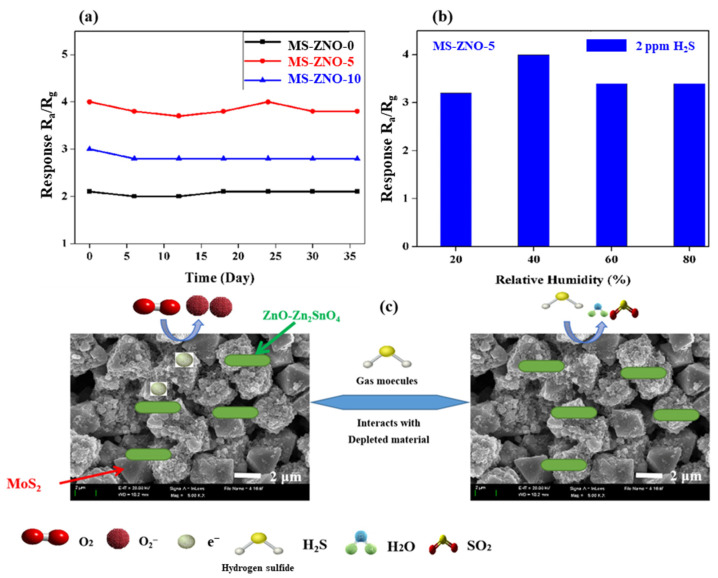
(**a**) The stability graph; (**b**) relationship between response of MS−ZNO−5 to 2 ppm H_2_S and different relative humidity at 30 °C; (**c**) diagram of gas sensing mechanism.

The stability evaluation in Figure 7a explained that the sensors of MS-ZNO-0, MS-ZNO-5 and MS-ZNO-10 were almost stable for approximately 35 days. The resistance of all the sensors decreased slightly with time. To date, the majority of the gas sensors based on semiconductor gas sensors were not satisfied, which demerits their applications in the field of gas sensors. However, in the present case we may see that the long-term stability was quite stable for almost a month. Different responses (3.2, 4, 3.4, and 3.4) to 2 ppm H_2_S were detected by the sensor of MS-ZNO-5 at various relative humidities (RH) of 20, 40, 60, and 80, respectively; the detail was revealed in Figure 7b. The highest response was detected at the 40% RH value. The results explained that the response was decreased after 40% RH due to the presence of the higher amount of water molecules with increased relative humidity; these can be adsorbed onto the surface of the material, and accordingly, the resistance decreases [41]. The results proved that the sensor based on MS-ZNO-5 was noteworthy in all aspects such as high response, humble selectivity, rapid response–recovery times, LOD, good stability, an almost linear relation between H_2_S gas concentration and response, etc.

### 2.3. Gas Sensing Mechanism

N-type gas sensing behavior was discussed based on the sensor of MS-ZNO-5 described in Figure 7c. Usually, all SMOs involve three steps, such as (1) adsorption; (2) oxidation; and (3) desorption. When the sensor of MS-ZNO-5 was tested in the air ambiance, the process was similar to our previous work [10]; first, oxygen molecules were adsorbed onto the surface of the material. There, they converted the captured electrons from the conduction band of ZnO-Zn_2_SnO_4_ into oxygen ions such as O^−^, O^2−^, and O_2_^−^, while forming the electron depletion layer and enhancing the sensor resistance, which was described in Equations (1)–(4). The adsorbed oxygen species depends on the operating temperatures, as shown in the following equations [42]. When the sensor of MS-ZNO-5 was tested in gas ambiance, the molecules of H_2_S reacted with oxygen ions and converted them into H_2_O and SO_2_, as mentioned in Equation (5). After that, the captured electrons were discharged back, due to the decrease in the resistance and space charge layer [10]. Thus, the sensor of MS-ZNO-5 showed a low resistance in gas atmosphere [43,44]. Further detail about the sensing mechanism has been given in the Equations below.

O_2 (gas)_ → O_2 (ads.)_, (1)

O_2 (ads.)_ + e^−^ → O_2_^−^_(ads.)_, T < 100 °C (2)

O_2_^−^_(ads.)_ + e^−^ → 2O^−^_(ads.)_, 100 °C ≤ T ≤ 300 °C (3)

O^−^_(ads.)_ + e^−^ → O^2−^_(ads.)_, T > 300 °C (4)

2H_2_S (g) + 2O_2_^−^ (ads) → 2SO_2_ + 2H_2_O + 3e^−^ (5)

Enhancement in gas sensing response of MS-ZNO-5 may correspond to some parameters due to the attachment of octahedron MoS_2_. Firstly, the formation of the n-n junction can also be one of the factors used to enhance gas sensing properties; the octahedron structure of MoS_2_ was checked by SEM and TEM [45]; the octahedron structure developed the exposure of active edge sites of MoS_2_, and improved the efficiency of gas transportation, reaction and carrier exchange, etc. This resulted in the enhanced H_2_S gas sensing properties and increased BET-specific surface area of MS-ZNO-5 (N_2_ adsorption–desorption isotherms); this factor can be very helpful to the adsorption and diffusion of H_2_S molecules. Moreover, the attachment with octahedron MoS_2_ increased the conductivity of the noteworthy composite [45] and enlarged the number of oxygen species (XPS), etc. This was also one of the parameters used to enhance their performance.

**Table 1:** The comparison of sensing properties between some sensors.

Materials	Temp. (°C)	Gas/Conc. ppm	Response (R_g_/R_a_)	Selectivity	Limit of Detection	Ref.
ZnSnO_3_	230	ethanol/50	47	1.4	1 ppm	[46]
ZnO/Co_3_O_4_	250	acetone/50	46	-	2 ppm	[47]
ZnO-ZnS	150	H_2_S/5	0.88	-	1 ppm	[48]
Pd/ZnO	220	CO/100	15	-	20 ppm	[49]
Zn_2_SnO_4_	133	H_2_S/1	-	-	1 ppb	[50]
Nb_2_O_5_/SnO_2_	275	H_2_S/20	4	3.8	-	[51]
Ag-In_2_O_3_	30	H_2_S/20	93719	-	0.005 ppm	[52]
MoS_2_-ZnO-Zn_2_SnO_4_	30	H_2_S/2	4	1.6	0.05 ppm	This work

Temp. = temperature, Conc. = concentration, Ref. = reference.

## 3. Experimental Section

### 3.1. Chemicals

The chemicals (molybdenum disulfide (MoS_2_), zinc nitrate hexahydrate (Zn(NO_3_)_2_·6H_2_O), tin chloride pentahydrate (SnCl_4_·5H_2_O) and sodium hydroxide (NaOH)) utilized in the synthesis method of ZnO-Zn_2_SnO_4_ nanoparticles and MoS_2_-ZnO-Zn_2_SnO_4_ nanocomposites were bought from the Sinopharm Chemical Reagent Limited Corporation (Dalian, China) and all these chemicals were used without further purification.

### 3.2. The Synthesis of ZnO-Zn_2_SnO_4_ Nanoparticles and MoS_2_-ZnO-Zn_2_SnO_4_ Nanocomposite

Hydrothermal was used to synthesize ZnO-Zn_2_SnO_4_ nanoparticles and MoS_2_-ZnO-Zn_2_SnO_4_ nanocomposites. Concisely, Zn(NO_3_)_2_. 6H_2_O (8 mmol, 1.477 g), and SnCl_4_.5H_2_O (4 mmol, 0.876 g) were mixed into three different beakers (1, 2 and 3) with 50 mL deionized water while stirring and then different contents of MoS_2_, such as 0 wt% MoS_2_ (MS-ZNO-0), 5 wt% MoS_2_ (MS-ZNO-5) and 10 wt% MoS_2_ (MS-ZNO-10), were added into beaker numbers 1, 2 and 3, respectively. After half an hour, 2M NaOH solution was gradually added to all the beakers to adjust the pH to about 12. The stirring process was carried out for 24 h to thoroughly mix all the ingredients and to receive the milky solutions. Furthermore, there was an autoclave process in which the samples were transferred into 100 mL stainless steel autoclaves and placed into an oven, and the time (20 h) and temperature (190 °C) were adjusted. After the autoclave process, the powder materials were separated by centrifugation process (washing with ethanol and DI water, 8000 rpm) and drying process (heating 60 °C, 20 h). Finally, the white products were calcined at 300 °C, 3 h and 5 °C/min. After that, the fabrication of gas sensors for gas sensing performances and other characteristics was carried out to check crystallite size, morphology and other properties of all the samples. The dried samples after calcination were ground in a mortar for fabricating the sensors and also for other characterizations such as XRD, SEM, TEM, etc. 

### 3.3. Characterizations of the Nanocomposites

Numerous characterizations have been used to identify the crystal size, BET-specific surface area, morphology and surface properties of synthesized products. Their deep explanation has been studied as follows: X-ray diffraction (XRD, D/MAX-Ultima, Cu Kα source, 2°/min scanning rate, scanning angle from 10°–80° as well as power of 40 kV and 40 mA, Rigaku, Tokyo, Japan), BET method (ASAP2010C instrument, Norcross, GA, USA), scanning electron microscopy (SEM, Suppa 55 Sapphire, Carl Zeiss AG, Jena, Germany), transmission electron microscopy (TEM, JEM-3200FS, JEOL, Tokyo, Japan), energy-dispersive X-ray spectroscopy (EDS, Sapphire 55 Supra, Zeiss, Jena, Germany) and X-ray photoelectron spectroscopy (XPS, Thermo Scientific ESCALAB 250 XI, ThermoFisher Scientific, Waltham, MA, USA) were used to check the crystal size, BET-specific surface area, morphology and surface properties of synthesized products. These tests were best performed by providing powder samples for XRD (about 20–30 mg of powder), SEM (about 10 mg of powder), TEM (about 10 mg of powder), EDS (about 10 mg of powder), BET (about 200 mg of powder) and XPS (about 5–10 mg of powder), respectively. 

### 3.4. Fabrication of Gas Sensor

**Figure 8:**
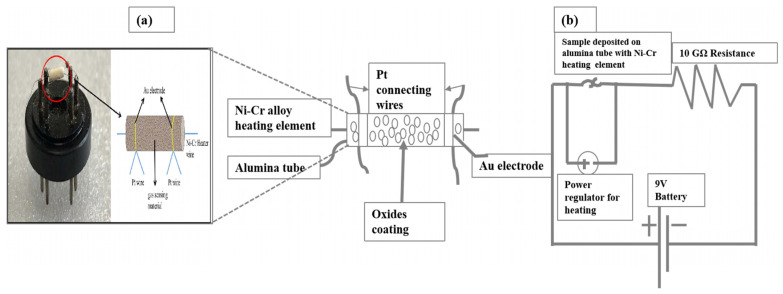
(**a**) The gas sensor device diagram and (**b**) the electrical circuit [8].

The gas sensor diagram and the electrical circuit were displayed in Figure 8. The fabrication process of the gas sensor was studied as follows: firstly, the paste was made with 0.2 g nanocomposite and 2–3 drops of terpineol and then the mixture was coated onto the outer surface of the alumina tube. After that, the alumina tube was heated in an oven for 10 h at 80 °C to remove the contents of terpineol. Then, a Ni-Cr alloy wire was placed in the alumina tube to control the operating temperature in the range of 30–400 °C. All the hazardous gases detected in the present work were bought from the Dalian Haide Technology Company Limited (Dalian China). The gas sensor based on MoS_2_-ZnO-Zn_2_SnO_4_ nanocomposites showed n-type gas sensor behavior and the response was calculated as S = R_a_/R_g_, where R_a_ was the resistance in air and R_g_ was the resistance in the gas. The selectivity of the sensor was calculated in this study, which may be defined as the ratio of the highest response and second highest response; in the present case, it was ‘S_10ppm H2S_/S_10ppm TMA_ = 1.6’, and likewise response/recovery times were stated as the time taken to reach 90% value of the final signal. The sensors were stable for 35 days and reproducible for five cycles.

## 4. Conclusions

The pure ZnO-Zn_2_SnO_4_ nanoparticles and nanocomposites were synthesized via a hydrothermal method. The synthesized materials were characterized using XRD, BET method, SEM, TEM, EDS and XPS, respectively. The XRD and SEM results may relate to each other, and we studied to see why the crystallite size and particle were increased when ZnO-Zn_2_SnO_4_ nanoparticles were attached with octahedron MoS_2_, respectively. From BET and XPS results, it was concluded that the MS-ZNO-5 nanocomposite revealed higher BET-specific surface area and more adsorption of oxygen species than MS-ZNO-0, which could be the main factors enhancing the gas sensing properties. The gas sensing properties of three gas sensors based on MS-ZNO-0, MS-ZNO-5 and MS-ZNO-10 were studied. The gas sensor based on an MS-ZNO-5 nanocomposite detected the highest response to 2 ppm H_2_S, and humble selectivity, rapid response/recovery time, good stability, promising reproducibility and LOD (0.05 ppm) were noticed. Furthermore, the sensor of MS-ZNO-0 and MS-ZNO-10 detected far fewer responses towards all gases. The enhancement in gas sensing response of MS-ZNO-5 corresponded with some parameters such as layered structure, n-n junction, higher BET surface area, more adsorption of oxygen species, etc. An almost linear relation between response and concentration of H_2_S (0.05–2 ppm) could allow the current sensor to be considered a potential candidate for gas sensing applications in the detection of and warning about leakage of hazardous VOCs.

## Data Availability

The data can be provided on the responsible request to the corresponding author.

## Sample Availability

Samples of the compounds are available from the authors.
